# Toward Patient-Centered AI Fact Labels: Leveraging Extrinsic Trust Cues

**DOI:** 10.1145/3715336.3735758

**Published:** 2025-07-04

**Authors:** Dong Whi Yoo, Austin M. Stroud, Xuan Zhu, Jennifer E. Miller, Barbara Barry

**Affiliations:** Indiana University Indianapolis, Indianapolis, Indiana, USA; Mayo Clinic, Rochester, Minnesota, USA; Mayo Clinic, Rochester, Minnesota, USA; Yale School of Medicine, New Haven, Connecticut, USA; Mayo Clinic, Rochester, Minnesota, USA

**Keywords:** Patient, AI Facts Label, AI Documentation, Trust

## Abstract

AI technologies in healthcare hold great promise for addressing numerous challenges, but ensuring that patients understand, trust, and adopt these technologies remains a significant hurdle. While the HCI community has proposed AI documentation frameworks (e.g., model cards) to enhance understanding, patient perspectives in the healthcare AI documentation remain underexplored. To address this gap, we designed prototypes based on existing frameworks and gathered feedback from 18 participants to explore their perspectives on AI documentation in cardiology, a domain where high-stakes AI tools are increasingly used and understanding users’ trust in AI is essential. Our findings revealed patient needs for more detailed information about healthcare AI technologies, the importance of extrinsic trust cues (e.g., regulatory status), and the integration of AI documentation into existing care processes. Based on these findings, we discuss two design implications: enhancing patient-centeredness in AI documentation and leveraging extrinsic trust cues to improve its design. This study contributes to the HCI community by amplifying the patient voice in designing AI documentation and offering actionable insights into leveraging extrinsic trust cues effectively.

## 1 Introduction

Artificial Intelligence (AI) enabled technologies are increasingly being used in healthcare [4, 31, 74]. As of August 2024, the United States Food and Drug Administration (FDA) has authorized 950 AI-enabled medical devices, a number that continues to grow rapidly [22]. Questions remain about “how, when, how not, and when not” to incorporate AI into practice [62]. To address these questions, Sendak et al. recently introduced the concept of model facts labels. A model facts label aims to provide frontline clinicians “with good information” on the risks of an AI enabled technology, to help them make “sound decisions [62].” While this is helpful, there is no such mechanism to inform patients on the risks and benefits of AI enabled medical technologies.

Concurrently, the human-computer interaction (HCI) communities have extensively explored strategies to ensure AI model fairness, accountability, transparency, and trust [2, 6, 15, 17, 46]. AI documentation, an emerging effort in providing relevant information about AI models such as model cards [49], has been proposed as one of the potential solutions to these challenges [3, 7, 17, 65]. Foundational studies such as model cards [49], factsheets [3], and datasheets [7] have inspired subsequent research on AI documentation frameworks. However, these existing frameworks often overlook the needs of end users of AI technologies, particularly in high-risk domains such as healthcare. This oversight may lead to unintended consequences: end users may lose autonomy in adopting AI technologies, place unwarranted trust in them, withhold trust due to insufficient information, or be marginalized due to unrecognized biases in AI models. Ultimately, these consequences can negatively impact patient health outcomes.

Specifically, we focus on patient perspectives in cardiology. Cardiovascular care presents an useful context to explore patient perspectives on designing AI Facts Labels, given the increasing integration of AI tools in high-stakes, risk-based clinical decisions, and the critical need to build trust through transparent, accessible communication about AI’s role in their care [40, 44].

Building on this focus, we designed a prototype of digital patient-centered AI documentation and solicited feedback from patient participants, incorporating the concepts of intrinsic trust (trust in the system) and extrinsic trust (trust in the stakeholders and social context) [32]. Our prototype design process and the feedback from patient participants suggested that the balance between intrinsic and extrinsic trust cues is essential to support patients’ trust judgment [42]. The feedback helped us better understand patient trust in AI technologies and the potential for AI documentation to facilitate this trust [47]. Specifically, our research questions were:

- RQ1: What are patients’ needs, concerns, and perspectives on trust in relation to AI documentation in cardiology care?
- RQ2: How can we design AI documentation in cardiology to be more patient-centered and trustworthy?

To address these questions, we synthesized existing AI documentation frameworks [3, 16, 49, 62] to design our prototypes. These prototypes were contextualized using vignettes describing the use of AI-enabled technologies in cardiology, and feedback was collected from 18 cardiovascular patients. Through reflexive thematic analysis [8], we identified three key themes: the need for clear information and labels, the importance of extrinsic trust cues, and the integration of AI fact labels into existing healthcare systems. Based on these findings, we discuss how patient-centeredness and extrinsic trust cues can inform the improved design of healthcare AI documentation. We contribute to HCI and the design of AI documentation by amplifying patient perspectives, which are often overlooked. This approach ensures that future AI documentation is more inclusive, user-centered, and aligned with the specific needs and concerns of patients.

## 2 Related Work

### 2.1 AI Documentation in HCI

As deep learning and machine learning gained momentum in the early 2010s, human–computer interaction (HCI) researchers have explored how we can document AI models to facilitate trust, transparency, accountability, and fairness. The ‘model cards’ framework, proposed by Mitchell et al., allows machine learning practitioners to report on their new models transparently [49]. The framework comprises nine sections, namely model details, intended use, factors (e.g., demographic groups), metrics (e.g., model performance measures), evaluation data, training data, quantitative analyses, ethical considerations, and caveats and recommendations. The sections on factors, ethical considerations, and caveats and recommendations are particularly important in ensuring the fairness of models, as they shed light on possible inequalities in outcomes for different demographic or cultural groups. This framework has inspired several practitioners and researchers, leading to follow-up studies that build on this concept. These include interactive model cards [16], practices for documenting machine learning models [5], and a community-centered authoring toolkit [64]. Crisan et al. created and assessed interactive model cards to assist domain expert analysts who lack AI expertise in comprehending and trusting AI models [16]. They elaborated on the model card framework by exploring interactivity and collecting feedback from those domain experts. According to the feedback obtained, when designing model documentation, it is crucial to take into account wider sociotechnical contexts and organizational dynamics. A model card authoring toolkit was designed and evaluated by Shen et al. in collaboration with Wikipedia editors and moderators [64]. Their objective was to scrutinize how members of online communities balance the trade-offs between AI model performance and community values, as well as to stimulate dialogues regarding their preferences. According to this conceptual study, community members acknowledge the needs of other concerned parties while evaluating trade-offs, highlighting the need for sociotechnical considerations to empower both communities and end-users. Based on valuable insights from model cards and subsequent studies, we emphasize the critical role of sociotechnical considerations for patients to utilize AI documentation effectively. We extend beyond previous studies on model cards by delving into the details of how these labels incorporate both technical aspects like functionality and performance, as well as sociotechnical information, including clinician recommendations and regulatory status.

Several researchers have explicitly focused on the datasets that underlie AI models regarding AI documentation [2, 7, 25, 55]. Gebru et al. drew inspiration from the electronics industry’s datasheets to introduce the concept of ‘datasheets for datasets’ aimed at enhancing transparency and accountability in the realm of machine learning [25]. Their joint efforts with academic and industrial partners have significantly influenced the community. The datasheets are tailored to the main stakeholders of this initiative, which are dataset creators and dataset users, mainly machine learning or computer science professionals. Pushkarna et al. introduced ‘data cards’ aiming to provide clear dataset documentation for stakeholders [55]. When developing these, they collaborated with a diverse group of stakeholders in the big tech industry to help them create responsible AI models. Although their intention was to extend the audience of data cards to include non-machine learning specialists, like UX or legal experts, they emphasized that their plan is not explicitly created for non-expert end-users affected by the resulting technologies. We build on prior research on datasheets and data cards to explore how non-expert end-users of healthcare AI products, specifically patients, understand AI documentation.

In summary, our study aims to advance the growing field of AI documentation in HCI by identifying design implications for patient-centered, end-user-focused AI documentation.

### 2.2 AI Documentation in Healthcare

The Food and Drug Administration (FDA) has utilized Drug Facts labels for over-the-counter (OTC) drugs in the United States (US) since 1999 [24]. Inspired by nutrition facts labels used in food regulation, drug facts labels are aimed at communicating usage and risk information to patients. Unlike Artificial Intelligence (AI) documents, drug facts labels use everyday plain language to communicate to their target audience. In recent times, medical researchers have introduced drug facts boxes [61], to extend drug facts labels to prescription medications. These boxes take it further by providing study findings and comprehensive information to assist patients in making well-informed treatment decisions. [62] proposed model facts labels that aim to aid frontline clinicians in understanding “how, when, how not, and when not to incorporate model prediction into clinical decision making [62].” The model facts labels point out vital categories for healthcare AI documentation, including intended uses, directions, warnings, mechanisms, and validation and performance. For example, the validation and performance categories include not only AUC (area under the curve), but also common clinical decision metrics like positive predictive value and sensitivity. Notwithstanding, the model facts labels did not take into consideration patient perspectives. Our research increases the range of the model facts label framework by investigating patient perspectives in the context of transparent healthcare AI documentation.

The primary motivation for this study is that existing AI documentation frameworks often fail to fully incorporate the perspectives of end users. In healthcare contexts, patient perspectives are particularly crucial for making AI documentation and systems more accessible, inclusive, and ethical. This aligns with the principles of patient-centered care [35, 71] and shared decision-making [27], frameworks that HCI researchers have actively explored. Patient-centered care and shared decision-making emphasize addressing the inherent power imbalances between healthcare systems and the individuals seeking help [20, 67]. These frameworks tackle ethical challenges by prioritizing the needs, values, and concerns of patients. Research has shown that such approaches not only increase patient satisfaction but also improve clinical outcomes [60]. Specifically, shared decision-making frameworks view patients as active participants in their care, capable of understanding the risks and responsibilities associated with treatments and contributing meaningfully to decision-making processes.

In addition to end-user perspectives, AI documentation must also engage with broader concerns around power imbalances and algorithmic bias. A growing body of research has shown that AI models trained on skewed or incomplete datasets can systematically disadvantage specific populations—such as racial minorities, older adults, or patients with rare conditions [14, 50]. To mitigate such harms, recent guidelines and scholarship emphasize the need for transparency in documentation to disclose how models were trained, who may be underrepresented in the data, and whether performance varies across demographic groups [1]. These fairness and equity considerations are not merely technical but also ethical and social, necessitating transparent fact labels that surface known biases, data gaps, and equity trade-offs. In this way, AI documentation serves not only to disclose the details of the technology but also as a tool for patient protection.

Our research builds on and extends the model facts Label framework [62] by centering patient perspectives in the design of healthcare AI documentation. Additionally, we posit that the healthcare domain is particularly pertinent for AI documentation. For several decades, HCI researchers have extensively investigated the healthcare domain [11, 26, 48, 53]. Similarly, Rostamzadeh et al. developed healthsheet, which is a health-specific application of datasheet [57].

In summary, our research draws inspiration from healthcare documentation practices, such as drug facts labels/boxes [24, 61], model facts labels [62], and healthsheets [57]. We aim to integrate AI documentation frameworks from HCI and healthcare to develop end-user-focused, patient-centered AI documentation for healthcare AI systems.

### 2.3 Intrinsic and Extrinsic Trust in Human-AI Interaction

In AI documentation, trust is a key element: its ultimate goal is to foster trust in the system, enabling users to adopt and use it effectively. With this in mind, we anchored our investigation of patient perspectives on patient-centered AI documentation in the concept of trust.

The HCI and Human-AI Interaction communities have made significant contributions to understanding trust in AI systems [37, 38, 43, 58, 69]. Emerging research has provided valuable insights into factors influencing user trust, including training data credibility [13], accuracy [73], explainability [21], and confidence representation [75]. While ‘trustworthy AI’ remains a nuanced concept, researchers have worked to define and contextualize it within human-AI interactions. For instance, Jacovi et al. formalized human-AI trust by emphasizing its contractual nature [32]. According to their framework, a trustworthy AI system fulfills its implicit or explicit obligations to users—for example, an AI-powered symptom tracker promises to monitor specific symptoms accurately. Trust is earned when the system meets these expectations, aligning its performance with its stated capabilities.

Trust in human-AI interaction can be categorized into two types: trust in the AI system itself, which determines whether users adopt and continue using it [16, 49], and trust in the decisions or recommendations generated by the AI system [31, 33, 39, 45]. While both are critical, trust in the system is more relevant to AI documentation, which typically serves as an onboarding or informational tool rather than explaining specific AI decisions or recommendations. Accordingly, in this study, we define trust as users’ willingness to adopt and continue using AI systems in cardiology, aligning with prior work that identifies trust as a key antecedent to acceptance and sustained use of AI-enabled applications in clinical care [66].

When considering how users can trust an AI system, two key concepts come into play: intrinsic trust and extrinsic trust [32]. Intrinsic trust arises from the inherent qualities of the AI system itself, such as its accuracy, reliability, and performance. Extrinsic trust, on the other hand, is shaped by external factors, such as the reputation of the organization behind the AI, regulatory approvals, or endorsements from trusted third parties. Together, these forms of trust influence how users perceive and interact with AI systems. While these concepts have guided researchers in exploring user trust and AI adoption [42, 69], they have not been fully applied to AI documentation, presenting a critical opportunity to expand this domain.

To better leverage the concepts of intrinsic and extrinsic trust in AI documentation, we designed prototypes that incorporated these trust cues. The prototypes categorized documentation items into intrinsic and extrinsic trust elements, aiming to explore how these concepts could enhance patient trust in healthcare AI systems. Our approach builds on prior research and extends existing AI documentation frameworks by integrating underutilized extrinsic trust cues.

## 3 Prototyping

### 3.1 Prototyping Process

The goal of our study is to gather patient perspectives on healthcare AI documentation. However, patients may not be familiar with healthcare AI or its associated documentation. To better understand their needs, preferences, and concerns, we designed a prototype of a patient-facing AI facts label. This prototype is intended to enhance healthcare experiences involving AI by addressing these considerations. Since patient-facing healthcare AI documentation is still in its infancy, our prototypes should be regarded as illustrative examples [62] or exploratory probes [30], aimed at deepening our understanding of this emerging concept.

Our label prototypes were designed to be displayed on mobile devices since patients widely adopt such devices, including smart-phones [54]. Moreover, a digital format enables updates with the latest healthcare AI information. We also envisioned that the same information can be provided in printed format for patients who lack personal mobile devices or in situations where a printed format may be more suitable.

We initially synthesized the current frameworks for AI documentation to develop the prototypes. Our process was guided by the following references: model cards [49], interactive model cards [16], datasheets [25], data cards [55], factsheets [3], and model facts labels [62]. The frameworks targeting AI practitioners and data scientists [25, 49, 55] informed the technical elements of the documentation. The frameworks aimed at service customers [3] provided insights into customizing documentation for patients inexperienced in AI. Based on these resources, we listed the potential elements and established criteria and contents for each.

In organizing items from the existing frameworks, we utilized the intrinsic and extrinsic trust cues suggested by Jacovi et al. [32]. Intrinsic trust cues refer to information that explains how AI models work, such as functionality, directions, and warnings, which were included in our prototypes. Extrinsic trust cues, on the other hand, pertain to how AI models behave, such as evaluation results. Jacovi et al. proposed three types of extrinsic trust cues: proxy (expert opinion), test sets, and post-deployment results [32]. Due to the emerging nature of many healthcare AI technologies, post-deployment results are often unavailable. To address this, we explicitly highlighted the absence of post-deployment data in our prototypes (e.g., “This device has not been evaluated in the real world.”). For test sets, we provided performance data based on the evaluation results of similar technologies. For proxy, or expert opinions, we included both clinician recommendations (recommendations from the patient’s clinical team) and regulatory status.

The label prototypes were created for two hypothetical technologies: Atrial Fibrillation (AF) Watch and Hypotension Prediction Index (HPI). AF Watch is an application that uses a smartwatch to detect potential atrial fibrillation, an irregular heart rhythm that can increase the risk of heart failure and stroke. The relevant AI-based smartwatch products [18, 70], including Apple Watch [51, 68], informed the development of our prototypes. HP Index, the second example, predicts potential hypotension, low blood pressure, by utilizing vital signs during surgical procedures [28]. We referred to the list of FDA-reviewed and authorized AI-enabled medical devices and software [36] when selecting our examples. We prioritized devices and software that offered ample publicly available information about their AI models—such as academic papers, clinical trials, and technical reports from manufacturers—to support transparency. Among the candidates, we selected AF Watch and HP Index because the former is a consumer-facing AI tool, while the latter is intended for healthcare professionals. This choice allowed us to capture a balanced range of perspectives from participants during the feedback-gathering sessions.

**Figure 1:**
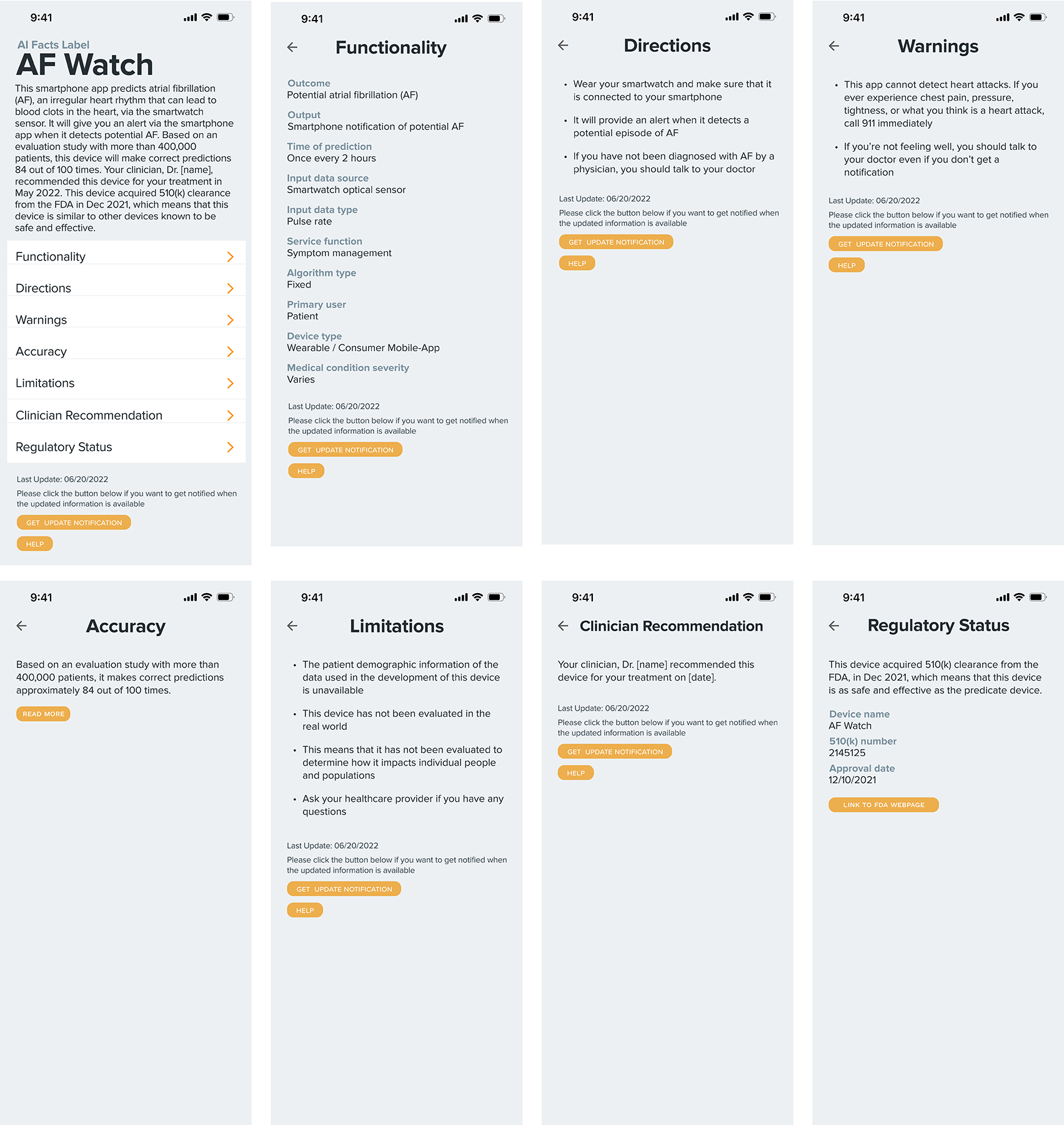
Prototype A (target technology: AF Watch). The AF Watch is a hypothetical product based on existing AI-based symptom checkers [19, 54, 75]. Patient participants reviewed this prototype using Figma during feedback-gathering sessions.

The early version of our prototypes was presented to two clinicians at a teaching hospital and three regulatory experts at a government agency. Two one-on-one remote meetings were held with clinicians at a large teaching hospital, and one group online meeting with regulatory researchers who specialize in reviewing AI-based healthcare devices and software. These experts provided invaluable feedback on the initial prototypes, recommending modifications to specific elements and advocating for the use of plain language. Clinicians assessed the appropriateness and suitability of the prototypes for presentation to patient participants in the upcoming feedback-gathering sessions. Meanwhile, regulatory researchers offered insights into the precise terminology used to describe regulatory status, such as 510(k) clearance, as shown in Figure 1.

Each element of the prototypes will be elaborated on in the next section.

### 3.2 Final Prototype

**Figure 2:**
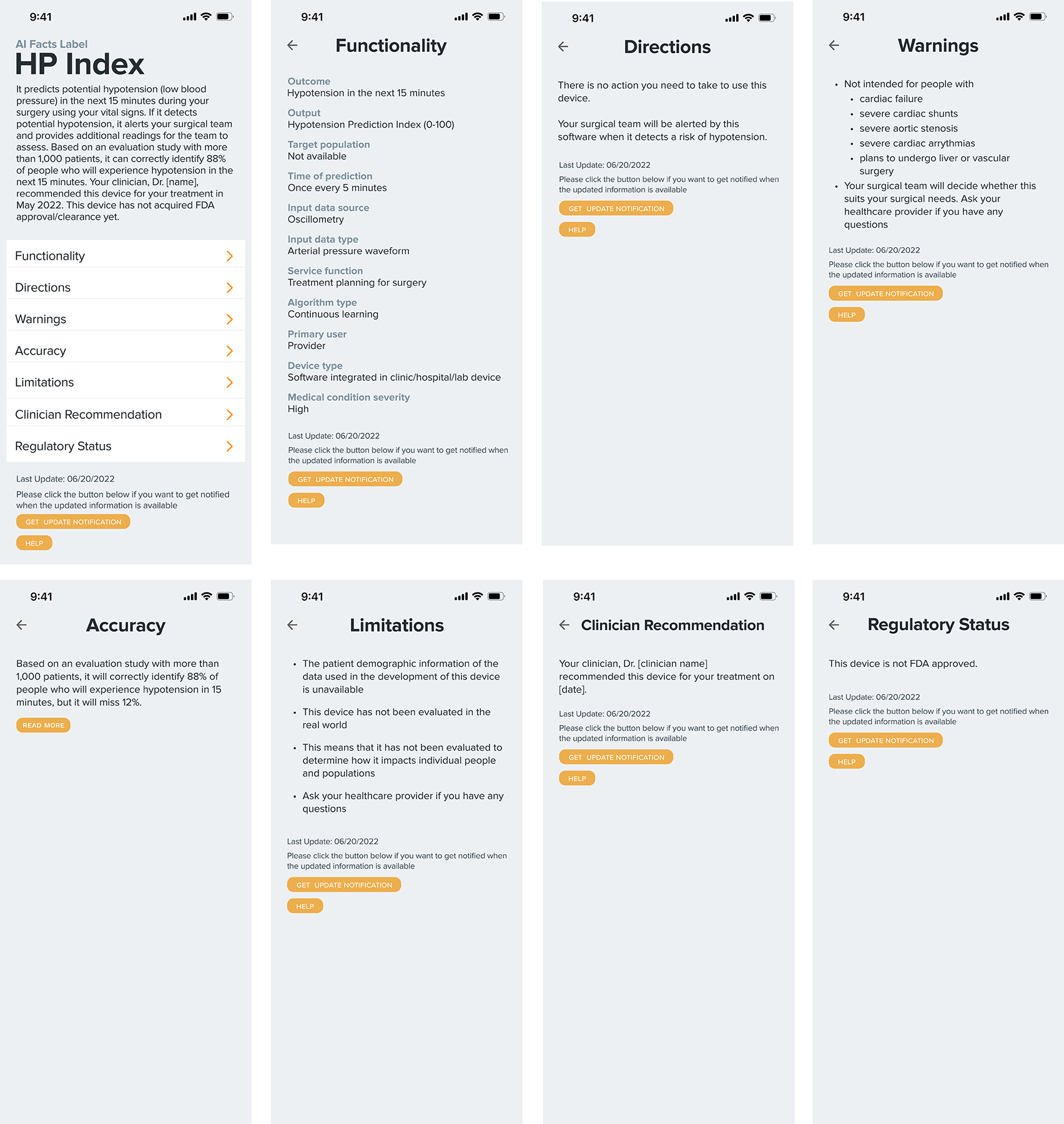
Prototype B (target technology: HP Index). The HP Index is a hypothetical product inspired by existing AI-based technology [30]. The main distinction between the HP Index and AF Watch (refer to Figure 1) is that the former is designed for clinicians, while the latter targets patients. Patient participants reviewed this prototype using Figma during feedback-gathering sessions.

This section provides detailed explanations of the prototype. Interactive labels for two AI-enabled healthcare tools were prototyped: AF Watch and HP Index. Each prototype comprises eight screens (see Figure 1 and 2), which will be further explained in the following subsections. The prototypes were created using Figma^1^, incorporating interactive functionalities such as clicking and swiping to navigate between screens. Several buttons, including the “Read More” button on the accuracy screen, were also interactive and expanded additional information when selected.

The **Home/Summary** screen is the first screen patients see, displaying an “AI Facts Label” header with the technology’s name (e.g., AF Watch or HP Index). It includes a brief summary explaining the technology’s purpose, mechanism, efficacy, and trustworthiness, highlighting clinician recommendations and regulatory backing. For instance, AF Watch is described as a smartphone app that predicts atrial fibrillation (AF) with an 84% accuracy based on a study of 400,000 patients, endorsed by clinicians, and cleared by the FDA. Below the summary, buttons link to sections such as Functionality, Directions, Warnings, Accuracy, Limitations, Clinician Recommendations, and Regulatory Status, allowing patients to explore more details. A footer indicates when the label was last updated and includes non-functional “Help” and “Get update notifications” buttons, which were discussed during the user study.

The **Functionality** section details the AI model’s operation, including its outcome, input data, algorithm type, and more [32]. Categories were adapted from existing AI documentation frameworks and refined based on feedback from clinicians and FDA researchers.

The **Directions** screen outlines patient actions required for using the AI technology, following established medical documentation practices [61, 62]. For example, AF Watch requires the patient to wear a smartwatch connected to their smartphone. For the HP Index, there is no action patients need to take because it is a clinician-facing technology.

The **Warnings** screen helps prevent misuse by outlining the technology’s limitations, such as AF Watch’s inability to detect heart attacks. It encourages patients to consult their clinicians with any concerns.

**Figure 3:**
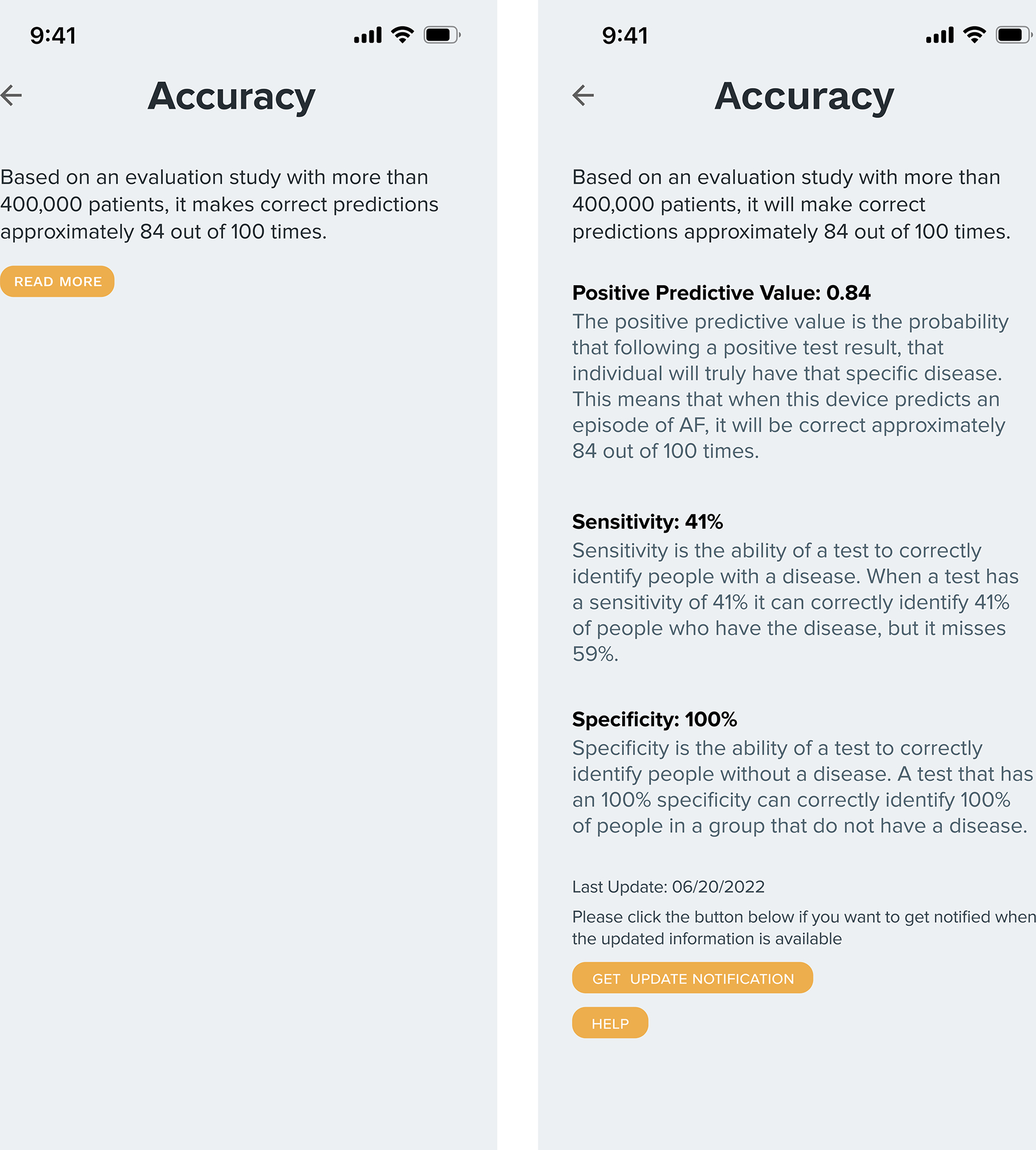
The Accuracy screens. When patients click the ‘read more’ button on the left, the screen will show more detailed performance metrics, as shown on the right.

The **Accuracy** screen presents performance metrics, including the positive predictive value (e.g., “correct predictions 84 out of 100 times”) and sample size [18, 28, 51, 70]. Additional metrics like AUC, sensitivity, and specificity are accessible via a “read more” button, catering to users with varying levels of technical comfort (see Figure 3).

The **Limitations** screen clarifies the constraints of the accuracy metrics, such as the lack of demographic data and real-world evaluation results. It highlights potential fairness issues and advises patients to consult their healthcare providers with questions.

The **Clinician Recommendations** screen indicates whether the technology has been endorsed by the patient’s healthcare provider. This transparency builds trust and aids in the decision-making process. This information also ties into the sociotechnical dimension (i.e., extrinsic cues [32, 42]) of healthcare AI facts labels.

The **Regulatory Status** screen provides information on the technology’s regulatory approvals, such as FDA 510(k) clearance [23]. For instance, AF Watch’s clearance in December 2021 is mentioned to indicate safety and effectiveness.

## 4 Feedback-Gathering Sessions

### 4.1 Procedures

#### 4.1.1 Recruitment and Participants.

The study recruited participants through ResearchMatch^2^, a national health volunteer registry created by several academic institutions and supported by the U.S. National Institutes of Health as part of the Clinical Translational Science Award (CTSA) program. ResearchMatch has a substantial number of volunteers who have given consent to be contacted by researchers about health studies for which they may be eligible.

The study recruited individuals aged 18 years or older residing in the United States who had primary care or cardiology health encounters in the past three years. We selected individuals with cardiology-related health issues because AI-based tools are increasingly being validated and used for diagnostics and treatment in cardiology, a field characterized by a high disease burden and diverse risks and interventions [40, 44]. This makes cardiovascular conditions particularly suitable for exploring patients’ perspectives on AI Facts Labels in healthcare. ResearchMatch delivered a recruitment message to potential participants that could either be affirmed, declined, or ignored. Participants who expressed interest in response to the recruitment message received a personalized invitation email and were scheduled for a remote one-on-one feedback gathering interview session. Participants who did not respond to the initial invitation email received up to two reminder emails.

Review and approval for this study and all procedures was obtained from the leading hospital’s Institutional Review Board. All participants consented orally to participate and have their interview digitally recorded.

We recruited 18 participants. Table 1 outlines the participants’ demographic information. The participants were compensated with a $25 gift card for their time and effort.

#### 4.1.2 Data Collection.

The first author conducted one-hour, one-on-one feedback-gathering sessions using the Zoom platform. At the outset of each session, the researcher covered the informed consent procedure with participants, and administered a demographic survey. This survey result is detailed in Table 1.

Subsequently, participants were asked about their interest in AI in general and whether they had prior experience with AI-enabled medical devices or software in their healthcare. These preliminary questions served as a warm-up, allowing participants to share personal experiences. Furthermore, they acted as an introduction to our prototype review.

Before presenting the prototypes, participants reviewed vignettes about a hypothetical patient user of the target technology. The purpose of these vignettes was to help participants situate themselves within the context of the target technologies [34]. The vignettes were developed by healthcare and HCI researchers in our research team and reviewed by clinicians and regulatory experts. The vignettes for the prototypes are shown in Table 2. The written vignettes were shared as text with the participants, except in one case where a participant struggled to read the text due to technical issue; in this instance, it was read aloud.

Prototypes were presented to the participants via Figma. A link to the Figma prototype was disseminated through the Zoom chat, allowing participants to access and interact with it using the web browser on their devices. The researcher could monitor cursor movements and any interactions participants had with the Figma prototype. Participants were encouraged to navigate each screen freely while adhering to a think-aloud protocol. Afterwards, they were prompted with questions about their initial reactions, interpretations of the screen, elements that clarified or obfuscated the target technology, factors that influenced their trust in the technology, and any other feedback or suggestions. In these discussions, trust was conceptualized as a factor shaping participants’ decisions to adopt and continue using the target technologies [16, 49]. We did not collect any quantitative measures of trust. Instead, we explored it when it emerged organically in participants’ responses to the prototypes, without directly prompting it.

Sessions ranged from 35 to 65 minutes in length. With the consent of the participants, sessions were recorded. These recordings were later transcribed by a professional transcription service and subsequently deleted.

#### 4.1.3 Data Analysis.

We utilized the reflexive thematic analysis as described by Braun and Clarke [8]. Throughout our data analysis, we endeavored to remain ‘reflexive,’ recognizing that our backgrounds in HCI, medicine, and public health might introduce unintended biases. We do not claim that our analysis represents all patient perspectives; rather, the primary aim of this study is to bring a subset of patient voices into AI documentation discussions. We hope future researchers will find value in our findings from a patient-centered design perspective.

The first and second authors began by immersing themselves in the data, reading the interview transcripts multiple times and sharing preliminary impressions with the rest of the research team. This familiarization phase helped the first author develop an interpretive sensitivity to recurring patterns, language, and tensions in the data.

Next, the first author conducted initial coding in Atlas.ti, a computer-aided qualitative analysis software. Coding was inductive and data-driven, focusing on semantic and latent meanings within participants’ responses [12]. In line with Braun and Clarke’s approach, the codes were not generated through line-by-line coding for quantification but through interpretive engagement with the data to capture salient features relevant to the research question [9]. This process yielded 43 preliminary codes.

As the analysis progressed, the first author iteratively revisited and refined the initial codes, merging overlapping ones, removing those that lacked analytic relevance, and deepening interpretive engagement with the data across transcripts. This reflexive process led to a more focused and conceptually rich set of 24 codes. Drawing from these, the first author generated a set of candidate themes by identifying patterns of shared meaning. These included: patients’ information needs, patient-AI-clinician interaction, non-AI-related questions, and patient concerns.

Throughout the process, the research team (including all authors) engaged in regular discussions to reflect on the evolving themes. We did not calculate inter-coder reliability, as reflexive thematic analysis does not treat coding as a mechanical act of consensus, but rather as a process of meaning-making where multiple perspectives are valued [12]. Instead, we used collaborative reflection to explore differences in interpretation and to deepen the analytic insights [9].

Through ongoing team discussions and further engagement with the data, we refined our themes into three overarching themes: needs for information and documentation, needs for sociotechnical cues, and the incorporation of AI Facts Labels into existing care systems. These themes represent interpretive, patterned responses across the dataset that illuminate participants’ perspectives on AI documentation in healthcare.

## 5 Findings

### 5.1 Needs for Information and Documentation

In the early part of the feedback-gathering sessions, we posed questions about participants’ prior experiences with AI in general and specifically in healthcare. Most participants had either heard of or used AI technologies in various forms, such as ChatGPT and recommendation algorithms on platforms like YouTube and other social media. Three of 18 participants reported encountering AI technologies during their healthcare treatment, particularly in robotic surgery and heart monitoring systems.

While robotic systems for surgery, such as the Da Vinci system, were not AI-driven at the time of this study but rather teleoperated tools controlled by human surgeons [41], participants in our study frequently described them as AI technologies. We present their accounts to reflect how patients perceive and mentally model automation in healthcare, which has direct implications for the design of patient-facing documentation.

Both P1 and P16 underwent surgeries assisted by robotic arms, but their experiences differed considerably. P16 underwent a laparoscopy involving the Da Vinci surgical robot [52]. His clinicians detailed why they opted for the robot: “She explained that the machine is designed to reduce errors, and they can more precisely identify the problem. The incisions and removals are also more exact.” Despite this, he felt apprehensive due to a lack of comprehensive information about how his clinicians would operate the robot. This led him to research and view related videos online independently.

“Well, at first, kind of nervous, ‘cause, you don’t see firsthand how they’re actually controlling the machine. I’m picturing like they’re on an Atari with a joystick [laughter]. […] Google, YouTube, videos. There’s always kind of something out there that allows you to have a visual of what’s happening. I did look at that as well to just kind of see how utilized the machine is. You know, how often is it something that gets used and percentage-wise of success, you know, and complications and stuff like that. There’s information out there other than just through my doctor’s office, so that was good too to be able to go and do my own sort of research on it to see how the machine itself actually works. […] Being able to see a video of it kinda helped as well to know what to expect for the procedure.”

–P16

Similar to P16, who felt more comfortable after watching videos about how clinicians operate the robot, P1 was reassured when his clinicians provided videos that detailed how robots would aid in his surgery. Echoing P16, P1 noted that the video alleviated much of his anxiety. P1 also highlighted the significance of knowing how frequently his surgeon and the affiliated hospital utilized the technology, as this information reinforced his trust in the process.

“Before the surgery, I had an appointment with the surgeon. They mailed me a packet detailing what would happen before the surgery, during the surgery itself, and the aftercare following the surgery. They also sent me a link, and it was a half-hour video. It actually showed the surgery. Not from inside the operating room, but it explained the whole procedure. A lot of the anxiety was relieved. It did show how the robot is used during the surgery. […] My surgeon has done over a thousand of these and has been with the hospital he’s affiliated with for about four years now. I have nothing but praise for robotics and surgery in the medical field.”

–P1

P12 was advised by his cardiologist to use a heart monitoring system. The instructions were to attach the device to the left pectoral area for ten days and press a button whenever he felt an irregularity in his heartbeat. The clinicians didn’t clarify that the device’s algorithm would automatically assess every detected heart rhythm and that his pressing the button was additional data to verify if he perceived the same irregular rhythm as the system. The inadequate instructions led P12 to believe he was solely responsible for recording his heart abnormalities; it caused him significant stress. He was concerned about potentially overlooking crucial signs or that his actions could lead to a severe diagnosis.

“What if I missed one, and what if it was the bad one? Then when I found out that, in fact, I didn’t have to ever hit the button if I didn’t want to––it would’ve still gotten caught. I would’ve felt so much better and I would like––I wouldn’t have been anxious. […] I was just very frustrated that they didn’t tell me and that I raised my anxiety needlessly.”

–P12

These narratives underline a recurrent perspective: patients independently seek more detailed information about the AI technologies incorporated into their treatments than what is offered by health care professionals during care. We probed what type of information they’d prefer regarding healthcare AI technologies if they were to be utilized in future treatments. Responses varied, encompassing topics like technology usage (P8, P15), risks and benefits (P7, P15), security (P8), privacy (P8), accuracy (P11), data usage (P14), and fairness (P18). Notably, several participants, even before reviewing our prototypes, referred to categories frequently discussed in existing AI documentation frameworks. Particularly, P18’s observations about potential biases align with the Model Card framework, which posits that AI models can sometimes introduce unintended biases.

“I’d like to know if someone reviews it, in terms of they’re just gonna go off with what the technology says and that’s the diagnosis or whatever they’re using it for. […] AI technology tends to be geared towards white people more than people of color, so I’d at least want to make sure that that’s something that they’re considering. For me personally, I guess that’s not really a big issue.”

–P18

After our participants reviewed our prototypes, we solicited their overall impressions and inquired if they’d prefer similar labels if AI technologies were used in future treatments. The consensus was favorable regarding our prototypes. Participants praised them for their organization, clarity, and user-friendliness. A few, like P1, emphasized the trust factor: “it builds a lot of trust in me in being comfortable and using the (AI-based) app.” Some, including P15, advocated for such labels to become a standard in healthcare AI.

“I think this needs to be a standard […] for any new AI technology because […] I’m not even aware of the chatbots and other things, AI or not. What happens to my data? How are they using it? Is it actually benefiting me or not? […] If you’re providing a new technology like AI, you have to provide the details and the facts of why you are using the technology and how. […] I just know that I’m informed about it. I think it needs to be a standard.”

–P15

However, we must note that a few participants, like P11 and P14, expressed a preference for oral explanations from clinicians over written labels. They argued that some patients might find these labels overwhelming or might misinterpret the information. This feedback suggests that while labels can be an informative tool, they shouldn’t replace the personal touch and reassurance that a direct conversation with a health care professional offers.

“I would probably rather just have the physician explain what’s going on instead of havin’ me read this label and trying to figure out what was goin’ on ‘cause I would probably misinterpret, especially when it says the severity thing. […] I think it’s just too much reading for a patient and too much to digest or understand, so I think maybe a short paragraph. […] I don’t know if you need all this stuff here.”

–P11

Given the points raised by P11 and P14 about the desirability of health care professionals to explain the label content, it is clear there is a balance to strike. While many participants also expressed a wish to discuss these matters with their clinicians, it’s important to note that healthcare AI fact labels are not intended to be the sole source of information. Instead, they should supplement the existing relationships patients maintain with their healthcare providers. This complementary role within the current healthcare system is further elaborated upon in Section 5.3.

To wrap up, participants expressed a clear need for detailed yet concise information about healthcare AI technologies used in their cardiology care. Specifically, they highlighted several categories of information as particularly useful for discussions with clinicians: the intended use and functionality of the technology (P8, P15), potential risks and benefits (P7, P15), accuracy P11), data privacy and security (P8, P14), as well as fairness and biases (P18). Participants mentioned that our label prototypes helped them better understand and trust these AI technologies by explicitly presenting this information. However, they also emphasized that such written documentation should complement, rather than replace, direct oral explanations and interactions with clinicians.

### 5.2 Needs for Extrinsic Trust Cues

During the feedback-gathering sessions, we asked participants to identify specific types of information that enhanced their trust in the target technologies. In this section, we discuss the categories of information frequently highlighted by participants. Notably, participants expressed a need for information that fosters extrinsic trust, which depends on external assurances or contexts [32], in the use of the target AI technologies.

Many participants stated that the details provided on the **accuracy** screen bolstered their trust in the target technologies. Some expressed that the purported accuracy of these hypothetical technologies was sufficiently high to merit their trust (P1, P2, P9, P11, P14, P15). Notably, the perception of what constituted high accuracy appeared to be subjective. However, P14 gave a specific threshold for her trust, remarking, “If it’s less than 75 percent accurate, I’m probably not going to trust it.” Others pointed out that the sample size in the evaluations boosted their trust in the target AI technologies (P5, P10, P11). For instance, we mentioned a sample size of 400,000 for AF Watch and 1,000 for HP Index in our prototypes. Yet, some participants had reservations about the provided accuracy data. Specifically, P3, P8, P12, and P14 expressed a desire to understand the implications of errors, such as false positives and false negatives. They felt that safety measures, like human collaboration to reduce mistakes, would enhance their confidence.

Several participants valued the **limitations** screen, appreciating its candor about what could and could not be verified (P10, P13, P17). P10 further expressed gratitude that the limitations screen prompted patients to discuss their concerns with clinicians. She said, “I like the honesty of it. It’s saying it sometimes doesn’t catch everything, but at least I’m aware. I’d be skeptical of something claiming 100% accuracy. This feels genuine. It’s a tool – not infallible, but a starting point for conversations with my physician.”

We deduce from these insights that our informants naturally connected accuracy and limitations. Given that the limitations screen elucidated unknown variables from the accuracy screen’s evaluation studies, our participants were positive about the technologies’ intentions. They indicated that if the technologies are accurate, they would be inclined to trust them.

Participants also highlighted the importance of regulatory review and approval and clinician recommendations. A significant number mentioned that the disclosure of whether a technology was approved or not either enhanced or undermined their trust in the target technologies respectively (P1, P5, P6, P9, P10, P11, P13, P15, P16). Prototype A indicated that the target technology (AF Watch) had received FDA clearance (510(k)), whereas prototype B noted that the target technology (HP Index) had not been authorized by the FDA. Our participants expressed a clear trust in the target technology of prototype A due to its regulatory status. Although many found the terminology (510(k)) challenging to understand, the overarching message that the technology had undergone FDA review instilled confidence. In contrast, there was skepticism towards the target technology in prototype B because it lacked FDA clearance.

“I would say regulatory status, because it has clearance from the FDA and safe and effective, so that would help my trust.”

–P6

“It worries me. I’m imagining you have to say this. This device is not FDA approved. As a patient, you’ve taken a lot of the wind out of my sails. That is to say you’ve gone backwards in credibility when I read that.”

–P13

It’s essential to clarify that we are not suggesting that patients should blindly trust or distrust approvals from regulatory bodies. We are highlighting that communication of the regulatory status of a device is highly impactful to users. Our findings emphasize the patient need for multiple layers of rigorous and trustworthy third-party reviews [58]. Patients should be supported to properly assess such certifications or approvals so that they do not make assumptions about the purpose and scope of regulatory guidance, such as assuming training data are representative or interpreting approval as a signal that the technology is low-risk or entirely without risk. Some participants, in fact, seemed to halt their consideration of the technology at regulatory approval rather than exploring a broader set of sociotechnical considerations.

Furthermore, informants identified the **clinician recommendation** screen as beneficial in establishing trust in the target technologies. As discussed in Section 5.1, participants expressed a desire to discuss AI technology information with their clinicians. Echoing this sentiment, P18 remarked that clinician recommendations and prior discussions with clinicians go “hand-in-hand.” Such information serves as both a reminder of previous conversations about technology usage and a prompt to revisit those discussions if necessary. P16 suggested that these recommendations felt personalized:

“I think a lot of it kinda links to that having, again, the statement of your clinician. Doctor so-and-so recommended the device. […] It actually is tailored towards the patient and gives them the data facts of what study was based on.”

–P16

In summary, our findings highlight that extrinsic trust cues, such as accuracy, limitations, regulatory approval, and clinician recommendations, play a critical role in fostering trust in AI technologies among patients. Participants valued transparency and candor, emphasizing the need for detailed yet comprehensible information about the capabilities and limitations of these technologies. While accuracy and regulatory approval often bolstered trust, participants also expressed a need for contextualized explanations of potential risks, such as false positives or negatives, and sought reassurance through clinician involvement.

### 5.3 Incorporating AI Facts Labels into Existing Care Systems

In Section 5.1, we discussed how some participants anticipated that their clinicians would verbally explain the information on our label prototypes. Meanwhile, in Section 5.2, we highlighted how some participants appreciated the limitations screen for prompting them to consult their clinicians with questions. In this section, we will explore another significant finding: participants expected that AI Facts labels would be integrated into the existing care workflow.

Several participants expressed how these labels could foster patient-clinician collaboration. For instance, P6 believed labels could streamline communication, stating, “The label is good. It might lead you to decide whether to consult your clinician or not. If you don’t need to, you save everyone time and money.” P10 shared her tendency to ask her clinicians health-related questions, including about AI technologies. P7 expanded on this, expressing hope that beyond the label, clinicians would advocate for the use of AI technologies as part of their duty:

“I think I would hope that my physician would say, ‘Listen, you’re at risk for this. […] I’m recommending that you wear this device and you monitor yourself all the time or when you’re out walking about.’ […] That’s what he’s there to do. He’s there to keep me up.”

–P7

Our participants provided insights into how these labels might be incorporated into clinical encounters. Given that the target technology of prototype B is used during surgery, participants imagined the labels might be distributed during presurgery consultations. Notably, some voiced a preference for a consent form at the conclusion of the labels, ensuring they approve of the AI-assisted treatment. A consent form, part of the informed consent process, is a document used by health care professionals and researchers to educate a patient about the risks, benefits, and alternatives of a proposed treatment, and by the patient to declare their voluntary participation in an intervention or study [63].

“For me, if it’s something like this, I would wanna have this printed out, and then I would wanna sign off on it. Almost that, ‘Yeah, I consent to using this in my treatment. I consent to the risk associated with how it’s going to predict it.”’

–P3

“I think it would be more useful to have it in the forms that the patient would review and sign before their surgery.”

–P9

By highlighting the consent procedure, participants communicated their desire for active involvement in clinical decision-making. They expect clinicians to detail AI technologies involved in procedures, discussing the risks and benefits. This aligns with the shared decision-making framework in healthcare, which emphasizes informed decisions and patient involvement. Clinical literature has shown that shared decision-making enhances patient-clinician relationships and improves clinical outcomes [19, 67].

Moreover, participants expressed interest in understanding how the tool is being used by clinicians and the explicit function of the AI in their care. They also wanted to know how clinicians compared to AI in symptom detection. Participants, including P5, also desired information on whether clinicians would oversee AI decisions:

“(if a clinician says) ‘There is going to be a highly skilled clinician who loves looking at data reading your results, and consulting with us if they see anything not good or nothing.’ That would definitely build my trust.”

–P5

P8 noted the need for information on handling false negatives and false positives, emphasizing that treatment decisions are ongoing processes. This suggests that future labels should outline clinician responses to AI inaccuracies.

“I’m just trying to think if 88 percent—having the exact statistics in there if it’s 88 percent accurate or not. That just leaves it open to questions about what happens to the other 12 percent. […] You don’t want to leave it where it’s having you ask other questions unless you’re gonna explain all that there too. That just makes it more untrustworthy.”

–P8

Considering the real-world application of AI technologies, some participants wanted information on insurance coverage. P11 stressed the importance of insurance coverage, highlighting it as a primary concern:

“Is this covered by insurance? […] If it—is it covered by Medicare? Is it covered by private insurance? […] That’s one of the first questions I would ask. Is there a monthly fee for this thing? How does this work? How is it reimbursable? I don’t know—I don’t see that answer anywhere.”’

–P11

In summary, this study captured patient perspectives on the integration of healthcare AI technologies into existing systems and the need for AI facts labels to consider issues beyond the clinical encounter. Participants expect AI labels to complement clinician input and to support shared decision-making (e.g., consent form). They also hope future labels detail the intricacies of clinician-AI collaboration and address concerns like insurance coverage.

## 6 Discussion: Implications for Patient-Centered AI Facts Labels for Sociotechnical Trust

We developed prototypes informed by existing AI documentation frameworks and gathered feedback from patients to better understand how they perceive intrinsic and extrinsic trustworthiness cues. Based on our findings, we propose two design implications: enhancing patient-centeredness in AI documentation and effectively leveraging extrinsic trust cues to improve its design. While our study focused on cardiology, we believe these implications hold relevance for healthcare AI documentation more broadly. However, we acknowledge that documentation needs may vary across clinical domains and stages of care, such as diagnosis, treatment, and prevention. Therefore, our proposed implications should be interpreted as foundational considerations that require further contextualization and refinement when applied to other healthcare settings.

### 6.1 Patient-Centeredness and AI Documentation

As we discussed in Section 2.2, our study builds on the principles of patient-centeredness [35, 71], and our findings resonate with shared decision-making frameworks [27]. Participants highlighted their need for more accessible information about AI technologies that might be used in their care. They also expressed interest in interacting with this information as part of their care routines. More importantly, participants identified value in both intrinsic and extrinsic information about potential AI technologies. This suggests that participants are not only interested in the practical applications of AI but also in understanding its technical aspects. We envision that patients will make more informed decisions about adopting and using AI-based tools in their care based on well-structured AI documentation.

However, patient autonomy in decision-making about adopting AI technologies raises important questions, especially for clinician-facing technologies. For example, in our prototypes and vignettes, the hypotension detection system is designed to be primarily used by clinicians during surgery. As researchers conducting exploratory work on healthcare AI documentation, we included this case to explore whether patients see value in learning about clinician-facing AI technologies. This raises a broader question: Should patients be informed about all technologies used by clinicians in their care?

Our participants shared that having more information about clinician-facing AI technologies helped them better understand their treatment and provided peace of mind. For example, P1 said that “a lot of anxiety was relieved” after reviewing a video explaining how robots support surgery. Similarly, P16 described searching for and watching videos about robot-assisted surgery “to know what to expect from the procedure.” Overall, our participants expressed an expectation that the clinical team would provide detailed information about their treatment, including AI-related technologies.

Based on insights from our participants, we advocate for providing patients with comprehensive yet carefully balanced information about AI technologies in healthcare. According to our findings, “comprehensive” information encompasses clear explanations of technology usage, accuracy levels, limitations, potential risks such as false positives or negatives, regulatory status, clinician involvement, and the frequency of technology use within clinical settings. At the same time, our findings also caution against overwhelming patients with excessively detailed information, as noted by participants P11 and P14, who preferred concise summaries combined with oral explanations from clinicians. Thus, striking the right balance involves providing key information clearly and concisely while facilitating opportunities for further patient-clinician discussion. This is especially important because AI technologies are still emerging, and their effectiveness and potential harms remain uncertain [72]. Ensuring that patients are informed about the use of these powerful tools aligns with principles of transparency, patient-centered care, and shared decision-making, ultimately empowering patients to actively participate in their healthcare journey.

### 6.2 Leveraging Extrinsic Trust Cues

The design of our fact label prototypes was informed by existing AI documentation frameworks, expert feedback, and the concepts of intrinsic and extrinsic trust. Jacovi et al. noted that intrinsic trust might not be achievable for lay users, even if the AI model is explainable, because intrinsic trust requires users to fully understand the model’s reasoning process and compare it to their own [32]. In healthcare contexts, it is particularly challenging for patients to develop their own reasoning processes for evaluating complex AI technologies. Unsurprisingly, our participants frequently mentioned specific types of information—such as limitations, clinical recommendations, and regulatory status—that we, as researchers, interpret as extrinsic trust cues. These cues appeared consistently in participants’ discussions as critical factors influencing their trust in the technology. In this section, we explore how extrinsic trust cues can be effectively leveraged in the design of AI documentation.

Jacovi et al. identified three sources of extrinsic trust: proxy, post-deployment data, and test sets [32]. Our prototypes incorporated all three: proxy in the form of clinical recommendations and regulatory status, post-deployment data in the form of limitations (acknowledging that healthcare AI technologies often lack comprehensive post-deployment results), and test sets in the form of accuracy data. Interestingly, we found that acknowledging less-than-perfect performance or a lack of evaluation can positively influence patient trust in AI systems. This highlights the importance of embracing transparency and acknowledging gaps in information or evaluation in future AI documentation.

In terms of proxy, which involves expert opinion on AI reasoning or behavior [32], healthcare AI presents a unique context due to the involvement of clinical teams and legal governance systems. These trust cues transfer trust from human-AI relationships to human-human or system-level relationships (e.g., trust in regulatory bodies), adding layers of complexity to AI documentation. However, this transfer poses risks of misplaced trust or mistrust. For instance, a patient with a positive relationship with their clinician might trust a system solely because of clinician endorsement, even if the system has limitations. Conversely, a patient with negative experiences in the healthcare system might reject a potentially beneficial technology due to distrust in the system as a whole. We believe that increasing transparency and providing more detailed information can mitigate these situations.

For clinician recommendations or endorsements, AI documentation should facilitate further discussions, both online and in person, to ensure patients are fully informed. Being fully informed means patients gain a clearer understanding of what an AI system does, its limitations, and how clinician judgment integrates with AI-generated insights. Our findings indicate that providing comprehensive and transparent documentation can increase patients’ trust and reduce their anxiety. Conversely, as illustrated by P12’s experience, inadequate documentation can significantly heighten anxiety, underscoring the critical importance of clarity and comprehensiveness in AI documentation. Furthermore, informed patients are more equipped to engage proactively in conversations with clinicians, as participants appreciated the labels prompting them to raise specific concerns or questions during clinical consultations. This engagement empowers patients by making them active participants in shared decision-making, thus enhancing their confidence in adopting AI-supported healthcare technologies. Regarding regulatory status, as our findings highlight, it is essential to develop more explainable ways of communicating this information. Beyond current practices, the development of additional extrinsic trust cues is crucial for improving healthcare AI documentation. For example, third-party auditing and certification [56] could serve as valuable trust signals, providing an additional layer of reassurance for patients.

### 6.3 Limitations and Future Work

Our work has provided valuable insights into patient-centered AI documentation in healthcare, but it is important to acknowledge its limitations. One notable limitation is the potential lack of representation among our participants, who may not fully reflect the broader demographic of cardiovascular patients. This concern arises from both the limited number of participants and their specific contexts within the United States. As our qualitative design research perspectives [29] and reflexive data analysis approaches [8] suggest, the goal of our research is to develop design insights that can inspire other researchers and designers while recognizing that we, as researchers, along with our data and the ways we interacted with it, may carry biases and limitations. Therefore, we encourage a cautious review of our findings and discussions when applying them in other contexts.

One such limitation is that the initial prototype development was shaped primarily by existing AI documentation frameworks, which reflect developer-centered perspectives. Although we sought to mitigate this by incorporating feedback from patient participants throughout the design process, we acknowledge that this approach may not have fully addressed the initial framing bias.

Therefore, we encourage a cautious review of our findings and discussions when applying them in other contexts. In developing our prototypes, such as those with accuracy, limitations, and clinician recommendations, we did not intend to be exhaustive. These prototypes were designed primarily as tools to facilitate the articulation of ideas and concerns by our patient participants. To ensure the safety and appropriateness of these prototypes, we incorporated feedback from clinicians and regulatory researchers. Our research utilized the FDA approval process as an example, but it is important to recognize similar efforts in other regions, such as those by the European Commission [10, 59] and that this instance is not an overall critique of regulatory agencies but rather a view into patient responses to regulatory status. The collaboration among international regulatory bodies, healthcare research, and patients is essential in addressing bias and promoting fairness in AI technologies.

Finally, a methodological consideration pertains to our use of interactive label prototypes rather than solely relying on semi-structured interviews to elicit patient feedback. We chose a prototype-based approach because it allowed participants to directly engage with tangible representations of AI documentation, enabling more concrete and nuanced feedback compared to abstract discussion alone. However, we recognize this method may have influenced participants’ perceptions by framing responses around predefined prototype elements, potentially constraining the breadth of ideas elicited. Future research might consider complementary approaches, such as combining prototypes with more open-ended interview formats, to further validate and enrich these findings.

## 7 Conclusion

This study explores patient perspectives on AI documentation in healthcare. First, we created AI documentation prototypes that incorporated the concept of sociotechnical trust-a blend of trust in the AI system and the surrounding stakeholders and context. Feedback from patient participants highlighted the need to balance both intrinsic (related to the AI system) and extrinsic (related to other stakeholders) cues to foster trust. Their insights deepened our understanding of how patients perceive trust in AI. A standout suggestion was the potential of AI fact labels for supporting shared decision making. This research brings a new perspective to AI documentation. Rather than limiting the focus to the AI model, as seen in other frameworks, our approach advocates that AI documentation for patients include non-AI-related data, such as insurance details, to provide a more complete picture. At its core, this paper highlights the urgent need for a holistic, patient-centric model for AI documentation in healthcare. Such a paradigm not only makes it easier for patients to justafiably trust and decipher AI systems, but also empowers them to make informed healthcare decisions.

## Figures and Tables

**Table 1:** Participant demographic information

ID	Age	Sex	Ethnicity	Home State (USA)
P1	64	Male	White	Connecticut
P2	64	Female	White	Wisconsin
P3	34	Female	Hispanic or Latino and White	Utah
P4	79	Female	White	California
P5	42	Female	White	Kentucky
P6	60	Female	White	New York
P7	76	Male	White	Ohio
P8	54	Female	White	North Carolina
P9	53	Female	White	Connecticut
P10	52	Female	Black or African American	Alabama
P11	65	Female	White	Virginia
P12	51	Transgender	White	Oregon
P13	74	Male	White	Indiana
P14	34	Female	White	Iowa
P15	25	Male	Asian	Utah
P16	47	Female	White	Minnesota
P17	75	Female	White	Tennessee
P18	24	Male	White	Massachusetts

**Table 2:** Vignettes used during the feedback-gathering sessions.

**Prototype A (AF Watch).** Nina just turned seventy and decided it is time to pay more attention to her heart health given that her family has a history of cardiovascular issues. She recently installed an app on her smartwatch that monitors her heart activity. The smartwatch uses artificial intelligence software that has been trained on half a million ECG recordings to identify atrial fibrillation. One day when she is out for a walk, the watch vibrates, notifying her that she is experiencing irregular heart rhythm. Nina uses the ECG feature of her smartwatch to take a reading, which returns a result that Nina is experiencing atrial fibrillation. Concerned with this classification, Nina calls the nurse helpline at her Primary Care Provider’s office to discuss the alert and follow up with any necessary tests. Nina also shares a copy of the ECG reading taken by her smartwatch with her Primary Care Provider by uploading it to her patient portal.
**Prototype B (HP Index).** A patient is undergoing surgery for acute appendicitis. While operating, the surgical team uses several devices to monitor the patient’s vitals. An artificial intelligence (AI) tool is analyzing the vital sign data to predict if the patient will have potential complications. The AI tool uses a model that is continuously learning from new data inputs from the vitals and outcomes of patients undergoing this procedure. The AI model updates based on what it learns on a regular schedule. During the surgery, the AI tool alerts the surgical team that the patient will likely experience hypotension and provides additional readings for the team to assess. The surgical team assesses the risk score and the state of the patient and proceeds to prepare additional intravenous fluids and blood transfusions to treat potential hypotension and prevent complications after surgery.

## CCS Concepts

- Human-centered computing → Empirical studies in HCI; Empirical studies in interaction design.

## Footnotes

1
https://www.figma.com

2
https://www.researchmatch.org/
